# Full-Scale Fire Smoke Root Detection Based on Connected Particles

**DOI:** 10.3390/s22186748

**Published:** 2022-09-07

**Authors:** Xuhong Feng, Pengle Cheng, Feng Chen, Ying Huang

**Affiliations:** 1 School of Technology, Beijing Forestry University, Beijing 100083, China; 2 School of Nature Conservation, Beijing Forestry University, Beijing 100083, China; 3 Department of Civil, Construction, and Environmental Engineering, North Dakota State University, Fargo, ND 58102, USA

**Keywords:** smoke root, static feature, dynamic feature, fusion, full scale

## Abstract

Smoke is an early visual phenomenon of forest fires, and the timely detection of smoke is of great significance for early warning systems. However, most existing smoke detection algorithms have varying levels of accuracy over different distances. This paper proposes a new smoke root detection algorithm that integrates the static and dynamic features of smoke and detects the final smoke root based on clustering and the circumcircle. Compared with the existing methods, the newly developed method has a higher accuracy and detection efficiency on the full scale, indicating that the method has a wider range of applications in the quicker detection of smoke in forests and the prevention of potential forest fire spread.

## 1. Introduction

For smoke detection in outdoor open spaces, smoke detection methods using chemical or optical sensors have limitations, as they are usually local sensors. On the other hand, in recent years, with the development of machine vision technology and the increasing investment in forest fire prevention in various countries, vision-based smoke detection methods have become more popular. With the assistance of vision-based forest fire monitoring, the number of fire occurrences, the affected areas, and property losses worldwide are decreasing every year [1]. However, the forest environment is complex, and there are many interferences, which are capable of inducing the false detection of fire. To reduce the false detection of fire and enhance the early warning systems of fire occurrence in forest, the detection of smoke, the most important visual phenomenon in the early stages of forest fires, with a high robustness and accuracy is urgently required [2].

Vision-based smoke detection methods can be divided into two categories, including the traditional and the deep learning methods. The traditional method involves classifying images using various image processing feature descriptors and calculations of these descriptors, mainly relying on the extraction of hand-crafted features, such as color [3], texture [4], and other information of the image. The common image processing methods include the optical flow method [5], wavelet energy [6], and background subtraction [7]. Meanwhile, the commonly used feature descriptors include the local binary pattern (LBP) [8], histogram of the gradient (HOG) [9], discrete wavelet transforms [10], and redesigned feature descriptors or improved feature descriptors. For instance, Liu et al. [11] proposed an LBP operator based on centrosymmetric gradient compensation, and Wang et al. [12] used the color and diffusion characteristics of smoke to define the time window and determined whether the smoke was generated by the slope of the fitting. For video smoke detection, the feature descriptors may also include the dynamic features between video frames [13].

The deep learning method automatically extracts features through a neural network after preprocessing the image. For image smoke detection, Zheng et al. [14] compared several target detection networks and found that EfficientNet has the highest average detection accuracy. For video smoke detection, Lin et al. [15] used 3D networks to detect smoke using video, and Ren et al. [16,17] made significant progress in their study on image dehazing with respect to fog. However, since fog is uniformly distributed within an area, while smoke, on the other hand, is randomly distributed, the detection methods should be different. As smoke detection using deep learning techniques requires a large amount of training data, this greatly limits its wide application. In addition, deep learning technology is still in the developmental stage; thus, it mainly relies on lower-level cues and rarely uses temporal cues or is compared with the manual methods.

As it is difficult to increase the accuracy of the existing traditional smoke detection methods and consider the universality of the hand-designed features, these methods often have a high false negative rate or high false positive rate. To address this challenge, Gao et al. [18] proposed using smoke roots as smoke features for smoke detection and developed a method based on fluid mechanics to detect smoke roots in videos. To adapt this method for long-distance scenes, Gao et al. [19] combined it with maximally stable extremal regions (MSER) to render the contour and shape of the smoke area more visible. Lou et al. [20] reduced the number of candidate smoke root points, therefore improving the computational efficiency of simulated smoke, but the detection speed still requires improvement.

The smoke root is an important feature for distinguishing smoke from other smoke-like objects. However, the existing smoke root detection algorithm is still under exploration, and both the detection speed and accuracy require further improvement. Generally, the challenge of detecting the smoke root involves accurately obtaining and defining the smoke root points. To solve this challenge, in this paper, we obtain the complete contour of smoke to calculate the exact root point of the smoke using a pixel-level fusion algorithm. More importantly, we redefine the smoke root and divide the video according to the distance between the camera and the place where the smoke occurs to account for the universal adaptability of the smoke root. Specifically, to more effectively detect the smoke roots, we develop a new smoke root detection method based on connected particles, which is insensitive to the distance between the smoke and the lens, avoiding false detection and missed detection caused by distance and improving the robustness of the scene change. The comparison between the newly developed method with the Gao’s method [19] indicated that the new method can significantly improve the speed of the detection of smoke roots.

## 2. Methodology

**Figure 1:**
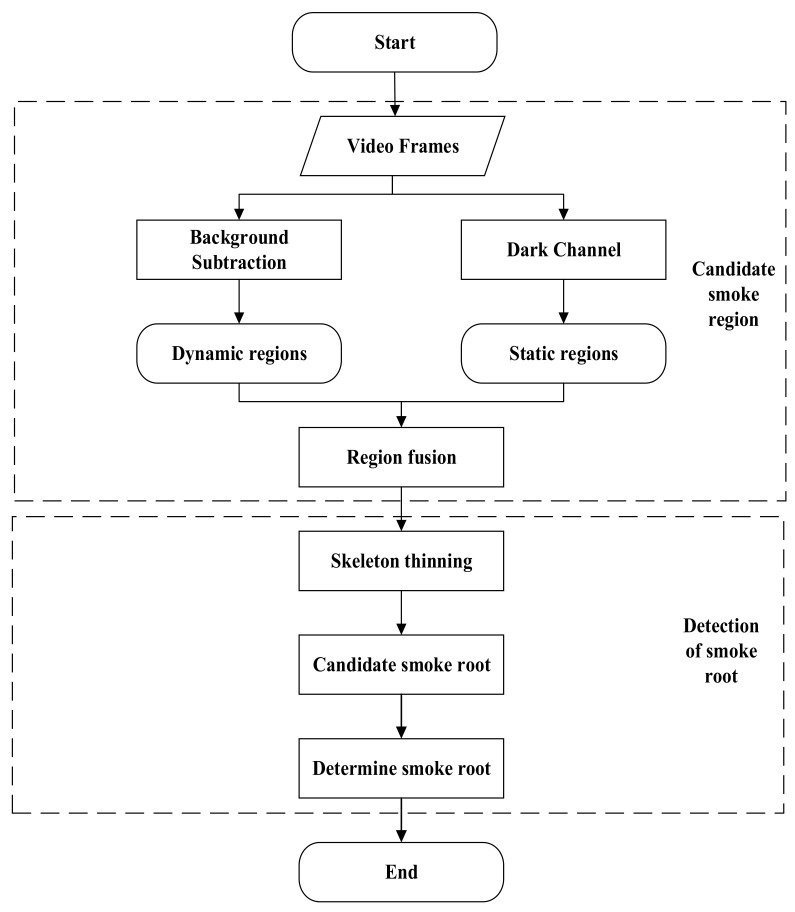
Flowchart of the proposed smoke root detection method.

As shown in Figure 1, the smoke root detection algorithm proposed in this paper mainly includes five stages: (1) dynamic candidate region extraction, (2) static candidate region extraction, (3) region fusion, (4) the extraction of skeleton points, and (5) the calculation of smoke points.

### 2.1. Dynamic Candidate Region Extraction

Since the background of the video used for forest fire monitoring does not usually change, when a fire occurs, moving objects, such as smoke, enter and form the foreground; thus, the background modeling method can be used to extract the smoke generated in the early stages of the fire. Existing background modeling methods include the CodeBook [21], SACON [22], and Vibe [23] methods. In practical scenes, the extraction of the dynamic regions may be disturbed by light, the movements of leaves, and weather. Thus, choosing an appropriate background modeling method is crucial for achieving the required detection accuracy. In this paper, background subtraction based on the Gaussian mixture model (GMM) method [24] is adopted for modelling the video sequence, which can effectively overcome the interferences of weather, leaves, or light.

### 2.2. Static Candidate Region Extraction

Through the extensive observation of fog-free outdoor images, He et al. [25] found that, in most non-sky local patches in haze-free images, at least one color channel has very low grayscale values at certain pixels. Considering that smoke and fog have similar color features, and that trees occupy most of the forest fire surveillance video, this color feature can be used to remove some interfering scenes in the video and reduce the false alarm rate by using the formula below:

(1) $$J^{dark}(x)=\underset{y\in \Omega (x)}{\min}[\underset{c\in [r,g,b]}{\min}J^{c}(y)]$$

where $J^{c}$ is the color for each channel, Ω(x) is the window x centered on the pixel, and $J^{dark}$ is the dark channel image. Thus, the binary image of a static candidate region (*static_img*) can be determined as:

(2) $$static\_img=\left\{\begin{array}{l} 0,\;\;\;\;\;if\;\;J^{dark}\to 0\\ 255,\;else \end{array}\right.$$

### 2.3. Region Fusion

**Figure 2:**
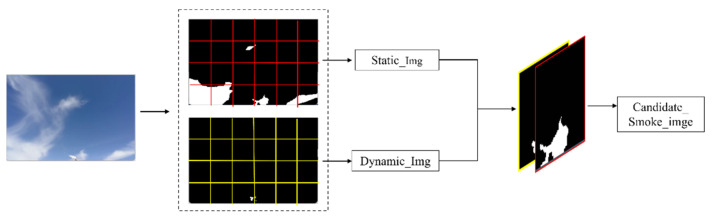
Fusion process.

The GMM algorithm can effectively detect smoke in short-distance and fast-changing dynamic regions, but it is easy to induce a false detection in long-distance and slowly changing dynamic regions. On the other hand, the dark channel algorithm can extract all areas that are similar to the color of the smoke, and there are no holes. To this end, we propose an image fusion method that combines the dynamic and static regions, as shown in Figure 2.

Since most of the same objects have the same color feature, a connected region may represent the same object, but there may also be objects with similar color features that are presented together in the scene, such as smoke and a house; thus, a connected region may also represent multiple objects. Therefore, smoke may exist in a single static connected domain or in a large connected domain. As shown in Figure 2, the fusion process firstly divides each frame of the smoke image into 10 × 10 grids and fuses the dynamic and static areas in each grid to avoid the phenomenon of “over-fusion”. Then, according to the position of the pixels in the dynamic area, it verifies whether the position corresponding to the binary image of the static area belongs to a static pixel point. If so, the point is defined as a candidate smoke point, and the current point is used as the center to determine whether its eight neighborhoods are static area pixels. This process is repeated for the points that meet the conditions until the grid area is exceeded. If it is not, the point is not defined as a candidate smoke point.

**Figure 3:**
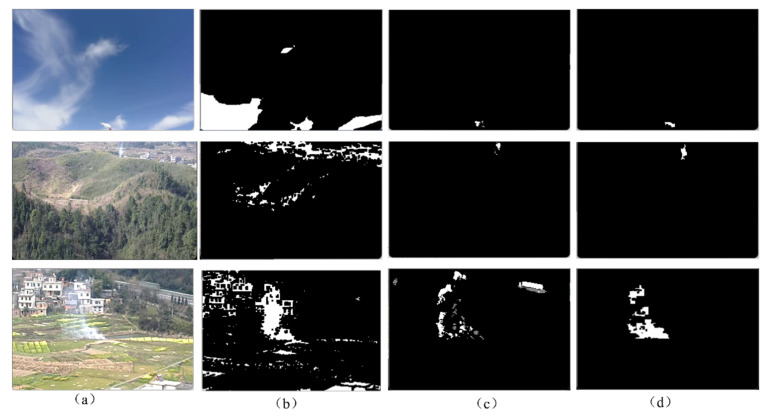
(**a**) Original image. (**b**) Dark channel image. (**c**) GMM image. (**d**) Fusion image.

Figure 3a–d shows examples of the original, dark channel, GMM, and fused images of forest fire smoke roots following the proposed fusion progress, as shown in Figure 2. Observing Figure 3a,b, it can be seen that the colors of clouds and buildings, such as houses and roads, are gray-white. Thus, in the process of static feature extraction, objects with similar color characteristics to smoke are extracted as distractors. However, regardless of whether the smoke is near or far away, it is completely extracted, and it may be integrated with the building. By observing Figure 3b,c, it can be seen that the GMM algorithm can only extract a small part of the smoke with no obvious change in the long-distance region, while more dynamic areas can be extracted for the smoke with an obvious change in the short-distance region, but in this case, the phenomenon of hollowing occurs. At the same time, since the cars on the road are also moving, they are extracted as interferences. Figure 3c,d shows that the fused image using the fusion process method developed in this paper not only fills the holes of the smoke to render the smoke area more complete, but also excludes the influence of vehicles, buildings, and clouds on the smoke detection.

### 2.4. Extraction of Skeleton Points

**Figure 4:**
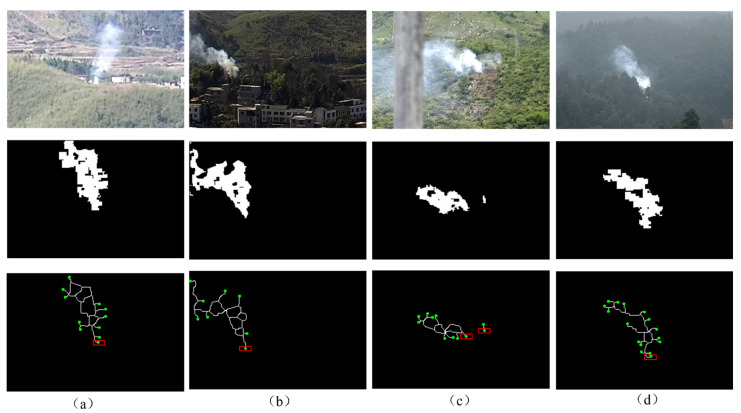
Original images (the first row), fusion images (the second row) and the smoke skeleton images (the third row).

In this study, the Zhang-Suen skeleton extraction algorithm [26] was used to extract the smoke skeleton. Each iteration was divided into two sub-iterations to remove the boundary and corner points of candidate smoke binary images. After many iterations, only the skeleton of the candidate smoke image remains. The second row in Figure 4 shows the fused images with smoke in four different distance environments, and the three row in Figure 4 shows the extracted skeleton images. From Figure 4, it can be seen that, in each scene, eight neighborhoods are detected. Accordingly, the endpoints of the smoke skeleton are identified, and the bottom endpoint is selected as the candidate smoke root node, which is marked with a red box in Figure 4. Among the objects, dynamic objects with a similar color to smoke are also marked.

### 2.5. Calculation of Smoke Root

**Figure 5:**
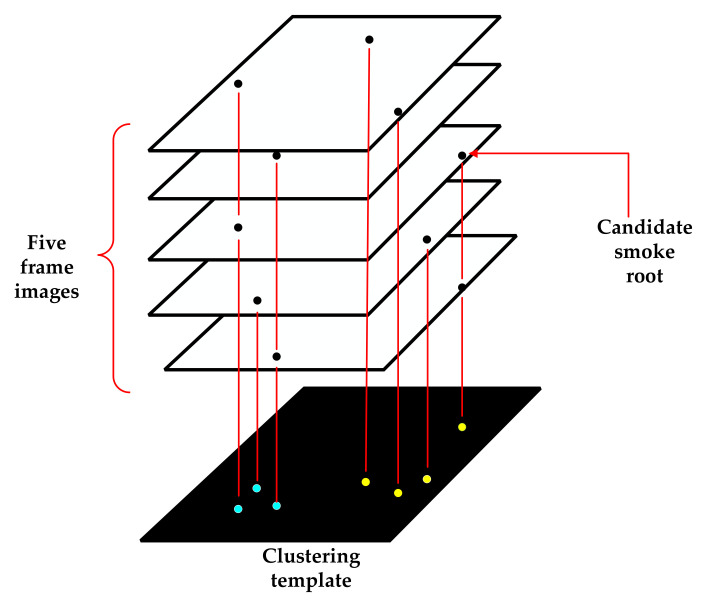
Smoke root matching.

As seen from Figure 4, there are still other objects causing interference when detecting the smoke. However, the important feature that can distinguish the smoke from these interfering substances is the “generalized root” of the smoke. Based on the definition of the smoke root by Gao [18], from a visual point of view, the “root” is not a certain pixel point on the image but a group of pixel points that is stable within a certain range. As the root is immobile, we can use the density of the candidate smoke root nodes to calculate the representative smoke root node and determine the smoke area, as shown in Figure 5.

**Figure 6:**
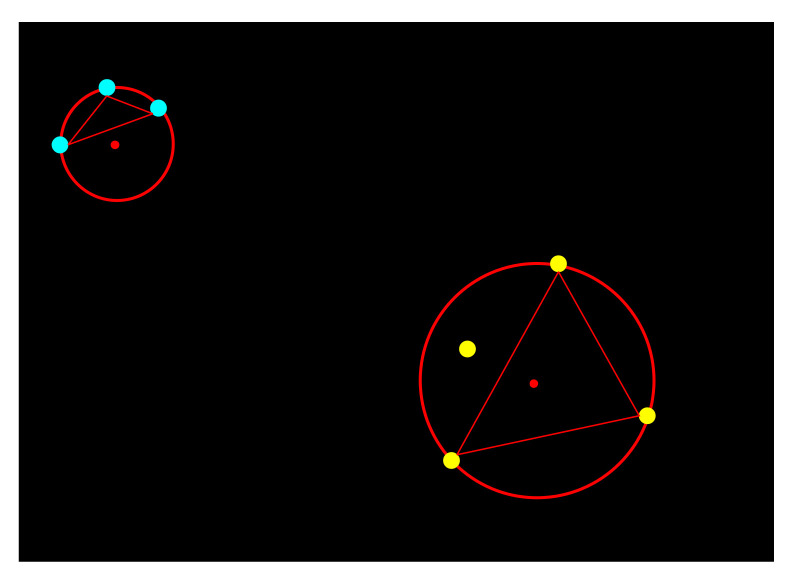
Circumscribed circle calculation.

As shown in Figure 5, firstly, five consecutive frames of candidate smoke root point binary images are stored in a queue, and then all candidate root nodes are projected onto the black template image. Finally, according to their density, the endpoints on the black template are processed to obtain a clustering template. After the clustering is complete, the number of endpoints of each category is obtained. The number of endpoints is classified as one of three categories, each of which corresponds to a different calculation method, as follows:

1. The total number is not more than three; thus, we exclude this area.
2. If the total number is greater than three, and the number of overlapping points is less than three, according to all the endpoint information of this type, we find the center and radius of the circumcircle, as shown in Figure 6. If the radius of the circumcircle is greater than the threshold, the area is excluded. Otherwise, the area represented by this category is a smoke area, and the coordinates of the center of the circumscribed circle are the coordinates of the node representing the root of the smoke.
3. If the total number is greater than three, and the number of overlapping points is greater than three, the most overlapping points represent the coordinates of the smoke root node.

## 3. Experiments and Discussion

### 3.1. Fire Smoke Video Dataset

**Figure 7:**
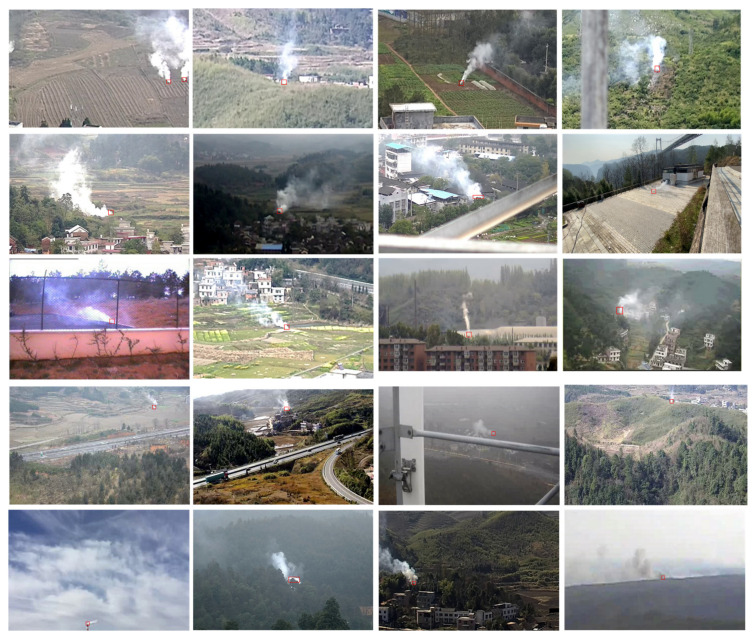
ROI areas for videos T1–T20.

To test the robustness and effectiveness of the developed algorithm, we selected 20 smoke sequences, some of which were gathered from public datasets on the internet, and the others were produced by the authors themselves, all of which were 480 × 320 in size. To validate the accuracy of the smoke root detection algorithm proposed in this paper [18], we established an artificial ROI area for each video. If the smoke candidate root was in the ROI area, the detection was determined to be successful; otherwise, the detection was deemed to have failed. Due to different scenes, the ROI sizes were also different. The specific ROI area size is shown in Table 1 and Figure 7.

At the same time, all videos were divided into long-distance and short-distance videos. ROIs greater than 10 × 10 were established as long-distance videos, and others were identified as short-distance videos. Among them, T1 to T12 are short-distance videos, where the smoke moving in the video is fast and occupies a large area, while T13 to T20 are long-distance videos, where the smoke is moving slowly and occupies a small area. As seen in Table 1, these 20 videos contain a variety of scenes and different background colors, including smoke surrounded by clouds, smoke obscured by pillars, and smoke that appears from houses in the evening.

### 3.2. Experimental Performance Analysis and Discussion

The smoke root detection method proposed in this paper was compared with two similar methods, including a forest fire smoke detection system based on the visual smoke root, which is a diffusion model proposed by Gao et al. (Method 1) [16], and a smoke segmentation algorithm based on improved intelligent seeded region growing (Method 2), proposed by Zhao et al. [27]. It is worth noting that, since the purpose of Zhao’s method is to detect smoke, not smoke roots, in order to compare the proposed method with Zhao’s method, we combined the developed smoke root node method in this paper with Zhao’s smoke detection so as to obtain the final results of Zhao’s method.

Table 2 shows the attributes of each test video used in this experiment, the total number of frames, and the times when the smoke roots first appeared (in frames) using the three different methods. If the smoke root was not detected, it is marked as “NO”. From Table 2, it can be seen that T1, T10, T14, and T17 were not detected by the other two methods. The common feature of these four videos is that there were disturbances similar to the color of the smoke in the scene, and the change in the smoke root is not obvious. In T17, especially, the cloud and smoke are almost integrated, which increases the difficulty of extraction. Method 1 places greater emphasis on the extraction of the foreground area, while the smoke changes slowly at the smoke source, meaning that this method is not ideal for such a scene. However, Method 2 segments the smoke using the method of region growth. For the parts with similar colors, over-segmentation easily occurs, resulting in the obtained smoke area being too large, and the position of the calculated smoke root node is offset. The method developed in this paper is applicable to such scenarios. In addition, although most of the smoke moves faster and is more easily detected in close-distance scenes, when viewing T4 and T9, we can see that none of the three methods detect the correct smoke root, the reason for which is that, in these two scenes, with the influence of the wind, the smoke in the video quickly spreads, and the real smoke root node is surrounded by the smoke, so that the real smoke root point cannot be detected.

According to Table 2, we calculated the detection accuracy of the methods for detecting smoke roots in the short-distance and long-distance scenes and the total smoke root detection rate. As shown in Table 3, it can be seen that Method 2 has only a 25% accuracy for long-distance scenes, while the accuracy of the proposed algorithm is as high as 87.5%. In short-distance scenarios, the accuracy of Method 1 and Method 2 are similar, at around 50%, while the method developed in this paper improved this accuracy by 25% to obtain a value of 75% compared with the other two methods. In general, the accuracy of the proposed method is significantly better than that of the other two comparison methods. Table 3 also shows that, for long-distance scenes, the method developed in this paper has a greater advantage, but for short-distance scenes, the advantages are not as clear as those for long-distance scenes. This is because, although the smoke occupies a large area in short-distance scenes, the proportion of the pixel group points of the smoke root in the whole smoke area is relatively small, and a complete smoke outline is required for the accuracy to be high. From Table 3, we can see that the accuracy rate of Method 2 for long-distance scenes is only half that for short-distance scenes, because the region-growing algorithm proposed by Zhao can accurately segment the smoke when the smoke occupies a large area. Thus, the accuracy of this method only amounted to 25% for the long-distance scenes in this experiment.

**Figure 8:**
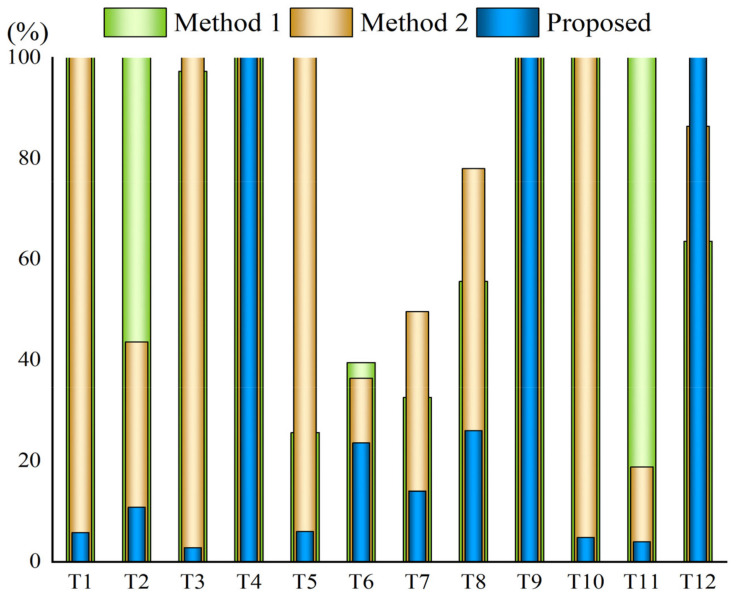
The proportion of time when the smoke root is first detected in the short-distance scene.

**Figure 9:**
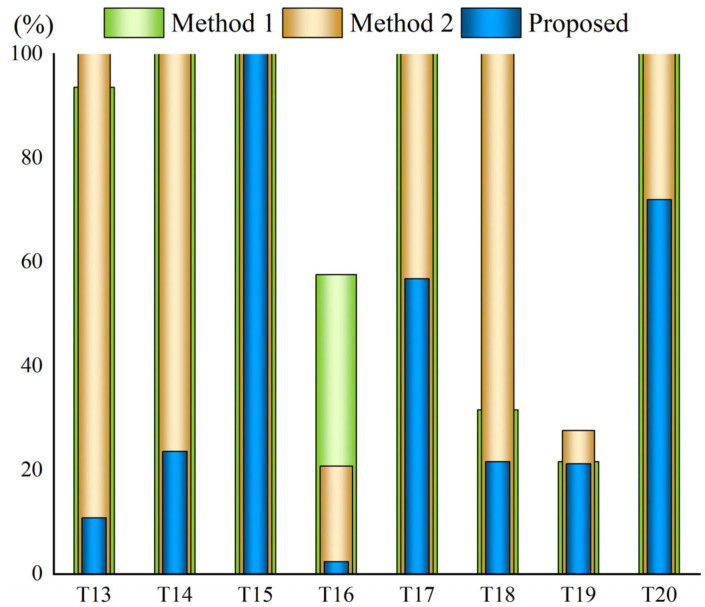
The proportion of time of the first detection of the smoke root point in the long-distance scene.

Figure 8 and Figure 9 illustrate the ratio of the number of frames to the total number of frames when the smoke root node is detected for the first time. If the ratio is 100%, it is considered that the smoke root point is not detected. As can be seen from Figure 7 and Figure 8, the method developed in this paper is more efficient. In short-distance scenes, the correct smoke root can often be accurately detected in the first 20% of the frames, followed by Method 1 and Method 2. For the long-distance scenes, the overall efficiency is lower than that of the short-distance scenes using all three methods.

**Figure 10:**
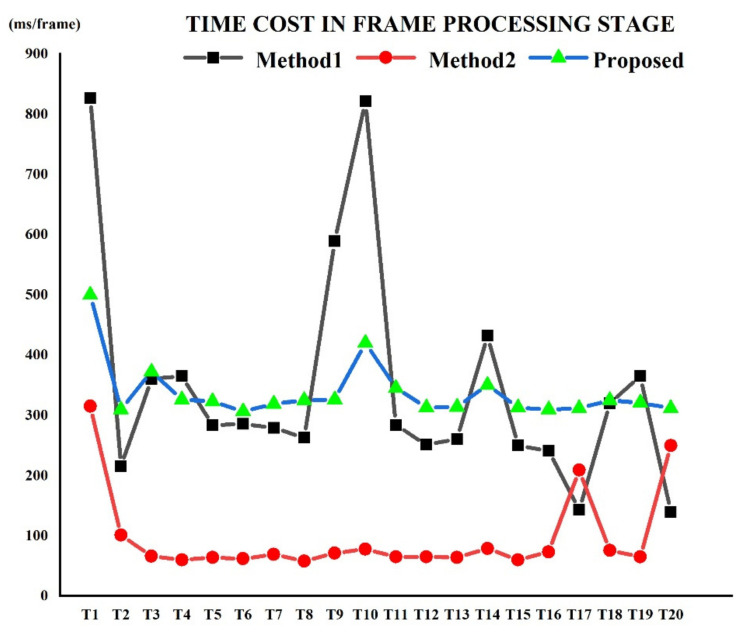
Average time cost in the frame processing stage.

As shown in Figure 10, the algorithm proposed in this paper is in the middle level in terms of time cost, but it is more stable. From Figure 8 and Figure 9, it can be seen that the method proposed in this paper has a high accuracy, and the accurate position of the smoke root points can often be detected at the early stage of the video, and even though the average processing time of each frame is longer, the overall efficiency is higher. The average efficiency of Method 1 is the lowest. The smoke area obtained by the Vibe algorithm used by Gao generally presents as multi-cluster scattered points, which need to be merged into a whole area through morphological operations, and the processed smoke outline expands outward, resulting in the offsetting of the boundary points of the smoke area. Moreover, the detection is performed every five frames within a group, delaying the detection rate. However, Method 2 places higher requirements on the test video. Under conditions where the smoke dynamic information is obvious and the colors of the scene in the video are quite different, the detected smoke area is more stable, while some test samples in this experiment show no obvious color differences. As the smoke moves slowly, a certain amount of time is required in order to detect the correct location of the smoke root. The fusion algorithm proposed in this paper can determine the root in five adjacent frames; that is, the position of the candidate smoke root can be calculated for each frame, which greatly saves time and space. The proposed fusion method can obtain a relatively complete smoke area, which is more conducive to the identification of the smoke root nodes.

## 4. Conclusions and Future Work

This paper proposes a new smoke root detection method that is not sensitive to the distance between the smoke and the lens by combining the GMM and dark channel prior algorithm to obtain the complete smoke area and improve the accuracy of the smoke root detection. In addition, we used the stability of the smoke roots to cluster the candidate points of the cigarette roots in five consecutive frames according to their density, obtained the circumscribed circle radius of the clustered points, and determined whether these were the final smoke roots, according to the circumscribed circle radius. The experiments showed that the newly developed smoke root detection method improves the accuracy by 37.5% to 62.5% in long-distance scenarios and, at the same time, the detection time is superior to that of the two existing algorithms.

The lack of datasets has always posed a serious problem for forest fire monitoring. Some researchers use synthetic or self-made methods in order to increase the number of datasets. However, the obtained data are too different from the real scene, resulting in a high false positive rate in practical applications. The question of how we can use a small amount of video material to accurately monitor forest fires is one of the problems that we need to solve. Moreover, future work may also consider how we can detect smoke in weather such as heavy fog and strong winds and further improve the performance.

## Figures and Tables

**Table 1:** Descriptions of the videos used for the experiments.

Name	ROI Size	Description
T1	12 × 12	Close-up of the smoke on the land with a grey background
T2	14 × 14	Smoke on the hillside with white buildings
T3	14 × 14	Smoke in a vegetable field with trees and houses of a similar color to the smoke
T4	14 × 14	Smoke on grass with large area disturbances, such as sticks
T5	14 × 14	Smoke in open space with distractors such as grass, trees, and dwellings
T6	14 × 14	Smoke rising over land in the evening, surrounded by trees and houses
T7	30 × 10	Smoke in a residential area, where most houses are similar in color to the smoke, and the video is blocked by a pillar
T8	10 × 10	wind, leaves, and people moving
T9	14 × 14	Smoke lit up on red ground with wind and moving people
T10	14 × 14	There are houses, roads, and moving cars
T11	14 × 14	Smoke from a factory building, and the whole picture is gray
T12	14 × 14	Smoke rising in a wooded area, with a few houses nearby
T13	10 × 10	Smoke from the side of the road, with moving cars
T14	10 × 10	Roads, cars, and mobile homes
T15	10 × 10	Thick fog, obscured iron railings
T16	10 × 10	Smoke on distant hillsides
T17	10 × 10	Smoke on the chimney, mostly slow-moving clouds
T18	10 × 10	Smoke rising from forest
T19	10 × 10	Position of the smoke at the foot of a mountain in the evening
T20	10 × 10	Smoke rising from flat ground in the distance

**Table 2:** Appearance times of the smoke roots.

Video	Distance	Total Frames	Method 1	Method 2	Proposed
T1	Short distance	225	No	No	13th
T2	Short distance	250	No	109th	27th
T3	Short distance	225	219th	No	6th
T4	Short distance	250	No	No	No
T5	Short distance	250	64th	No	15th
T6	Short distance	225	89th	82nd	53rd
T7	Short distance	250	84th	124th	35th
T8	Short distance	250	139th	195th	65th
T9	Short distance	250	No	No	No
T10	Short distance	250	No	No	12th
T11	Short distance	250	No	47th	10th
T12	Short distance	250	159th	216th	No
T13	Long distance	250	234th	No	27th
T14	Long distance	250	No	No	59th
T15	Long distance	250	No	No	No
T16	Long distance	250	144th	52nd	6th
T17	Long distance	250	No	No	142nd
T18	Long distance	250	79th	No	54th
T19	Long distance	250	54th	69th	53rd
T20	Long distance	250	No	No	180th

**Table 3:** Test results.

	Short-Distance Accuracy	Long-Distance Accuracy	Total Accuracy
Method1	50%	50%	50%
Method2	50%	25%	40%
Proposed	75%	87.5%	80%

## Data Availability

Not applicable.

## References

[1] Cruz H., Gualotuña T., Pinillos M., Marcillo D., Jácome S., Fonseca C.E.R., Botto-Tobar M., Cruz H., Díaz Cadena A. (2021). Machine Learning and Color Treatment for the Forest Fire and Smoke Detection Systems and Algorithms, A Recent Literature Review BT—Artificial Intelligence, Computer and Software Engineering Advances.

[2] Geetha S., Abhishek C.S., Akshayanat C.S. (2021). Machine Vision Based Fire Detection Techniques: A Survey. FIRE Technol..

[3] Mahmoud M., Ren H. (2019). Forest fire detection and identification using image processing and SVM. J. Inf. Process. Syst..

[4] Zhao Y., Zhou Z., Xu M. (2015). Forest Fire Smoke Video Detection Using Spatiotemporal and Dynamic Texture Features. JECE.

[5] Wu Y.L., Chen M.H., Wo Y., Han G.Q. (2021). Video smoke detection base on dense optical flow and convolutional neural network. Multimed. Tools Appl..

[6] Zhang Y., Wang H., Fan X. (2020). Algorithm for detection of fire smoke in a video based on wavelet energy slope fitting. J. Inf. Process. Syst..

[7] Vijayalakshmi S.R., Muruganand S. Smoke detection in video images using background subtraction method for early fire alarm system. Proceedings of the 2017 2nd International Conference on Communication and Electronics Systems (ICCES).

[8] Tang T., Dai L., Yin Z. Smoke image recognition based on local binary pattern. Proceedings of the 2017 5th International Conference on Mechatronics, Materials, Chemistry and Computer Engineering (ICMMCCE 2017).

[9] Qin L., Wu X., Cao Y., Lu X. (2019). An effective method for forest fire smoke detection. J. Phys. Conf. Ser..

[10] Wu X., Cao Y., Lu X., Leung H. (2021). Patchwise dictionary learning for video forest fire smoke detection in wavelet domain. Neural Comput. Appl..

[11] Liu Z., Yang X., Liu Y., Qian Z. (2019). Smoke-detection framework for high-definition video using fused spatial-and frequency-domain features. IEEE Access.

[12] Wang H., Zhang Y., Fan X. (2020). Rapid early fire smoke detection system using slope fitting in video image histogram. Fire Technol..

[13] Jia Y., Chen W., Yang M., Wang L., Liu D., Zhang Q. (2021). Video smoke detection with domain knowledge and transfer learning from deep convolutional neural networks. Optik.

[14] Zheng X., Chen F., Lou L., Cheng P., Huang Y. (2022). Real-Time Detection of Full-Scale Forest Fire Smoke Based on Deep Convolution Neural Network. Remote Sens..

[15] Lin G., Zhang Y., Xu G., Zhang Q. (2019). Smoke detection on video sequences using 3D convolutional neural networks. Fire Technol..

[16] Ren W., Zhang J., Xu X., Ma L., Cao X., Meng G., Liu W. (2019). Deep Video Dehazing With Semantic Segmentation. IEEE Trans. Image Process..

[17] Ren W., Pan J., Zhang H., Cao X., Yang M.-H. (2020). Single Image Dehazing via Multi-Scale Convolutional Neural Networks with Holistic Edges. Int. J. Comput. Vis..

[18] Gao Y., Cheng P. (2019). Forest fire smoke detection based on visual smoke root and diffusion model. Fire Technol..

[19] Gao Y., Cheng P. (2021). Full-scale video-based detection of smoke from forest fires combining ViBe and MSER algorithms. Fire Technol..

[20] Lou L., Chen F., Cheng P., Huang Y. (2022). Smoke root detection from video sequences based on multi-feature fusion. J. For. Res..

[21] Kim K., Chalidabhongse T.H., Harwood D., Davis L. (2005). Real-time foreground-background segmentation using codebook model. Real-Time Imaging.

[22] Wang H., Suter D. (2007). A consensus-based method for tracking: Modelling background scenario and foreground appearance. Pattern Recognit..

[23] Barnich O., Van Droogenbroeck M. (2010). ViBe: A universal background subtraction algorithm for video sequences. IEEE Trans. Image Process..

[24] Zivkovic Z., Van Der Heijden F. (2006). Efficient adaptive density estimation per image pixel for the task of background subtraction. Pattern Recognit. Lett..

[25] He K., Sun J., Tang X. (2011). Single image haze removal using dark channel prior. IEEE Trans. Pattern Anal. Mach. Intell..

[26] Zhang T.Y., Suen C.Y. (1984). A fast parallel algorithm for thinning digital patterns. Commun. ACM.

[27] Zhao W., Chen W., Liu Y., Wang X., Zhou Y. (2019). A smoke segmentation algorithm based on improved intelligent seeded region growing. Fire Mater..

